# *Transcriptomics, metabolomics*, and *in-silico* drug predictions for liver damage in young and aged burn victims

**DOI:** 10.1038/s42003-023-04964-2

**Published:** 2023-06-02

**Authors:** Beata Malachowska, Weng-Lang Yang, Andrea Qualman, Israel Muro, Devin M. Boe, Jed N. Lampe, Elizabeth J. Kovacs, Juan-Pablo Idrovo

**Affiliations:** 1 Department of Radiation Oncology, Albert Einstein College of Medicine, Bronx, NY 10461 USA; 2 Department of Surgery; Division of G.I., Trauma, and Endocrine Surgery, University of Colorado, Aurora, CO 80045 USA; 3 Graduate Program in Immunology, University of Colorado, Aurora, CO 80045 USA; 4 Department of Pharmaceutical Sciences, Skaggs School of Pharmacy, University of Colorado, Aurora, CO 80045 USA; 5 Molecular Biology Program, University of Colorado, Aurora, CO 80045 USA

**Keywords:** Mechanisms of disease, Transcriptomics, Gene regulatory networks

## Abstract

Burn induces a systemic response affecting multiple organs, including the liver. Since the liver plays a critical role in metabolic, inflammatory, and immune events, a patient with impaired liver often exhibits poor outcomes. The mortality rate after burns in the elderly population is higher than in any other age group, and studies show that the liver of aged animals is more susceptible to injury after burns. Understanding the aged-specific liver response to burns is fundamental to improving health care. Furthermore, no liver-specific therapy exists to treat burn-induced liver damage highlighting a critical gap in burn injury therapeutics. In this study, we analyzed transcriptomics and metabolomics data from the liver of young and aged mice to identify mechanistic pathways and in-silico predict therapeutic targets to prevent or reverse burn-induced liver damage. Our study highlights pathway interactions and master regulators that underlie the differential liver response to burn injury in young and aged animals.

## Abstract (web summary)

In a mouse model of skin burn, transcriptomics and metabolomics data reveal that the liver plays a role in the differential outcomes of young and aged mice.

## Introduction

Over 450,000 people are affected by burn injuries yearly, resulting in 40,000 hospital admissions in the United States alone^1^. Although there have been significant advances in burn injury therapeutics, burns are still a leading cause of morbidity and mortality worldwide^2^. Burns extend beyond the skin, inflicting damage on distant organs and exacerbating the already poor outcomes in burn victims, particularly when multiple organs experience failure^3^. Thus, deepening our understanding of how different organs respond to burns can help identify targets to direct organ-specific treatments and improve the outcomes of burn patients.

The liver is among the critical organs affected after burns, displaying significant morphological alterations, such as hepatomegaly and fatty infiltration^4^. Compared to controls, liver weight increases significantly 2 to 7 days post-burn in rodents^5^. Burn-induced liver damage (BILD) is associated with edema formation and at the same time may lead to cell damage and the release of hepatic enzymes^5^. Liver enzymes, such as aspartate aminotransferase (AST) and alanine aminotransferase (ALT), are sensitive indicators of hepatocyte injury^6^. Clinical evidence reveals that serum AST and ALT peaked during the first week post-burn^7^. Because the liver orchestrates metabolic functions, inflammatory processes, and immune events during basal and stress conditions, a burn patient with an impaired liver is more vulnerable to metabolic and infectious complications leading to unfavorable outcomes than one with a healthy liver^4^. Gong et al. studied the mortality risk of burn patients finding that the survival rates were significantly lower in burn patients with elevated liver transaminases than in those with normal values^8^. Liver damage after burns has been associated with multifactorial events; hypoperfusion, proinflammatory cytokines, cell death signaling, edema, fatty changes, and mitochondrial dysfunction^5^. However, BILD pathophysiology is not entirely understood, highlighting a critical gap in burn injury knowledge.

Using a clinically relevant murine burn injury model, our laboratory and others demonstrated that the liver exhibits molecular and histological changes following cutaneous burns^9–11^. Also, we showed that these alterations were exacerbated in aged animals, as noted by elevated liver injury markers, increased lipid deposits, heightened oxidative stress, and accentuated histological evidence of hepatic damage^10^. These observations highlight changes in the liver of burn-aged animals that indicate increased susceptibility of this organ to acute stressors.

Comprehensive genetic and metabolic studies can help elucidate the nature of the differences in liver response to burns between young and aged individuals. While transcriptomics determines the functional response to burn injury and helps predict its master regulators, metabolomics provides a downstream and closer to phenotype description of the biological processes that occur in the liver^12,13^. Here, we deepened the study of hepatic parenchymal cellular interaction by analyzing transcriptomics and metabolomics in the liver of mice after burn injury.

Additionally, we employed an in silico analysis of the databases obtained from the transcriptomics and metabolomics to predict drugs that might prevent or treat the liver pathology observed in mice after burn injury.

## Results

### Overview of the unsupervised analysis among the experimental groups

**Fig. 1:**
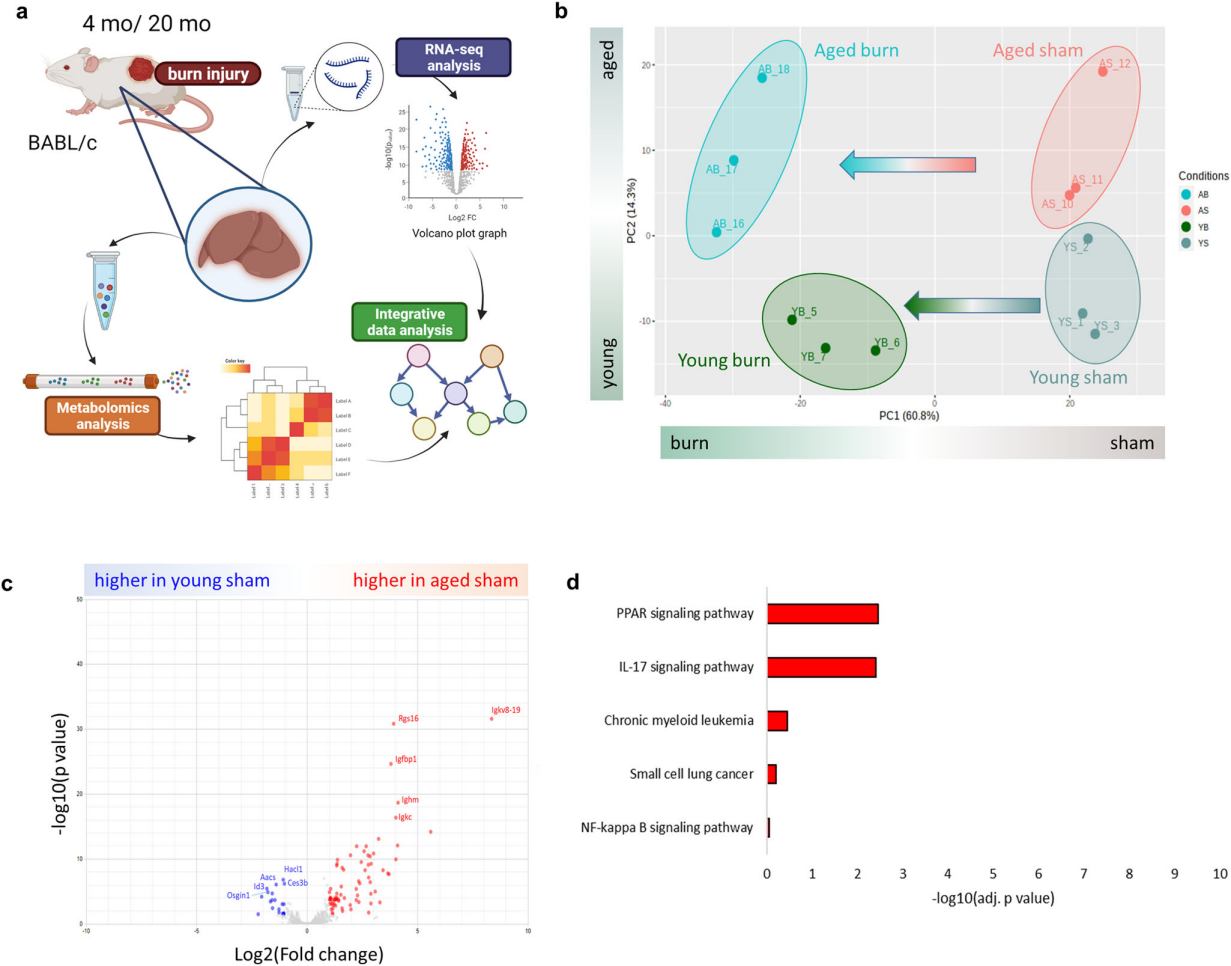
Experiments overview. **a** Experimental mice model. **b** PCA (Principal Component Analysis) overview of sample clustering. **c** Volcano plot showing differential liver gene expression between aged and young mice in sham conditions (without burn). **d** KEGG pathway overrepresentation analysis of genes up-regulated in aged mice. *N* = 3 per group.

Four groups of liver samples from the young sham, young burn, aged sham, and aged burn groups were collected 24 h after the burn injury. This time point was selected based on previous animal experiments showing systemic differences, specifically in the liver, between young and aged mice after burn^10,14^. Transcriptional analysis was performed by RNA-seq and targeted metabolomics with mass spectrometry (Fig. 1a). The samples maintained an adequate group separation observed in unsupervised analysis by principal component analysis of transcriptomic data (Fig. 1b). The first component identified and separated the sham and burn treatments (PC1 = 60.8%). The second component separated young and aged groups (PC2 = 14.3%).

### Hepatic transcriptomics in young and aged sham animals

At first, we focused on characterizing the differences between aged and young sham groups, finding that 75 genes were up-regulated (log2FC > 1 and adj. *p* value < 0.05) in aged compared to young mice (Fig. 1c). Among those, 13 genes were related to immunoglobulin production, including *Igkv8-19, Ighm, Igkc, Igkj5*, and *Igkj1* (Supplementary Data 4). In overrepresentation analysis, the genes up-regulated in aged mice were associated with two pathways, “PPAR signaling” (*Pck1, Angptl4, Plin5, Cyp4a32, Cyp4a14*) and “IL-17 signaling” (*Cebpb, S100a9, Nfkbia, S100a8, Cxcl1*) (Fig. 1d, Supplementary Data 7). There were only 20 down-regulated genes (log2FC < −1 and adj. *p* value < 0.05) compared to the aged vs. young mice after sham treatment. No pathway was statistically over-represented among down-regulated genes (Supplementary Data 8).

### Hepatic transcriptomics, shared response to burn injury by young and aged mice

**Fig. 2:**
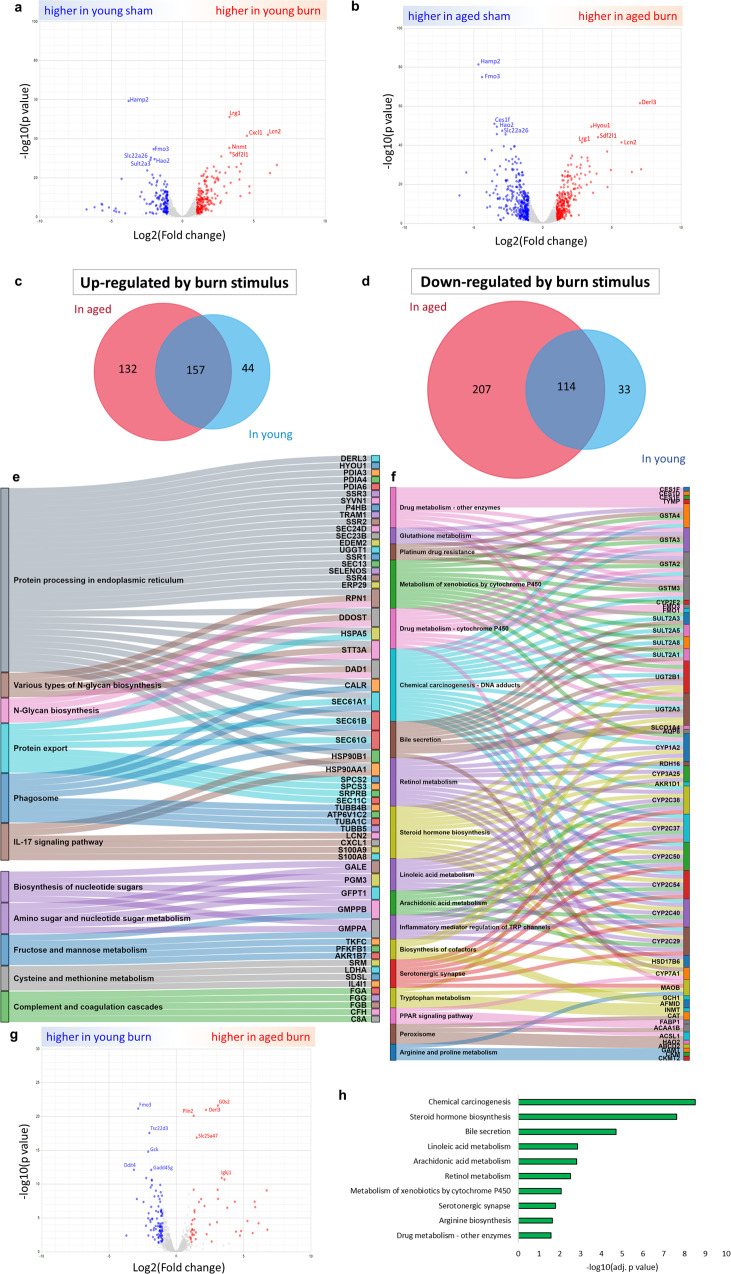
RNA-seq analysis of liver burn response in young and old mice. **a** Volcano plot showing genes responding to burn stimuli in young mice. **b** Volcano plot showing genes responding to burn stimuli in aged mice. **c** Venn diagram showing overlap of genes with up-regulated expression by burn stimuli in both aged and young mice. **d** Venn diagram showing overlap of genes with down-regulated expression by burn stimuli in both aged and young mice. **e** Sankey diagram of KEGG pathway from overrepresentation analysis based on overlapping up-regulated genes (panel C). **f** Sankey diagram of KEGG pathway from overrepresentation analysis based on overlapping down-regulated genes (panel D). **g** Volcano plot showing differential liver gene expression between aged and young mice after burn stimuli. **h** KEGG pathway overrepresentation analysis of genes down-regulated in aged burn mice vs young burn mice. For panels **e**, **f** and **h**, only pathways meeting the following criteria were shown. Size term < 200, intersection > 3, Benjamini–Hochberg adj. *p*-value < 0.05. *N* = 3 per group.

Furthermore, we identified a common hepatic transcriptomic profile of burn-injury response in both young and aged animals. We identified genes responding to burn stimulus from the differential expression analysis in young (Fig. 2a) and aged (Fig. 2b) groups and selected the ones shared between the age groups. In the up-regulated genes after burn injury, 157 genes overlapped between young and aged groups (Fig. 2c). Those genes were associated within the 11 KEGG pathways like “protein processing in the endoplasmic reticulum” (30 genes, e.g., *Derl3, Hyou1, Pdia3, Pdia4, Pdia6*), “protein export’ (8 genes identified, e.g., *Hspa5, Sec61a1, Sec61b, Sec61g, Spcs2*),” fructose and mannose metabolism” (5 genes: *Tkfc, Gmppb, Gmppa, Pfkfb1, Akr1b7*) and “IL-17 signaling” (6 genes: *Lcn2, Hsp90b1, Cxcl1, Hsp90aa1, S100a9, S100a8*) (Fig. 2e, Supplementary Data 5). In the down-regulated genes, there were 114 genes that overlapped in both young and aged groups (Fig. 2d). These down-regulated genes were significantly overrepresented in 18 KEGG pathways, including “chemical carcinogenesis – DNA adducts” (18 genes, e.g., *Sult2a3, Gsta4, Cyp3a25, Ugt2b1, Gsta3*), “steroid hormone biosynthesis” (13 genes, e.g., *Akr1d1, Hsd17b6, Ugt2a3, Cyp2c38, Cyp2c37*) and “metabolism of xenobiotics by cytochrome P450” (12 genes, e.g., *Gsta2, Cyp1a2, Sult2a8, Cyp2f2, Sult2a1*) (Fig. 2f, Supplementary Data 6).

### Hepatic transcriptomics, differences in response to burn injury by young and aged mice

After burn, there were 46 up-regulated genes and 89 downregulated genes in the livers of aged mice compared to their younger counterparts (Fig. 2g). No pathway was significantly overrepresented among the up-regulated genes (Supplementary Data 9). However, among down-regulated genes, we found eleven overrepresented pathways, including: “chemical carcinogenesis” (11 genes, e.g., *Gsta3, Gsta4, Sult1a1, Cyp2c54, Cyp2c38*),” steroid hormone biosynthesis” (10 genes, e.g., *Akr1d1, Hsd3b3, Cyp2c40, Cyp2b10, Cyp2c29*), “bile secretion” (8 genes, e.g., *Abcb11, Nr0b2, Hmgcr, Atp1b1, Sult2a5*), metabolism of linoleic acid, arachidonic acid and retinol metabolism (5 common genes - *Cyp2c54, Cyp2c38, Cyp2c40, Cyp2c29, Cyp2c50*), “arginine biosynthesis” (*Gpt, Gls2, Ass1*), and” metabolism of xenobiotics by cytochrome P450” (*Cyp2f2, Gsta3, Gsta4, Sult2a5, Sult2a8*) (Fig. 2h, Supplementary Data 10). We observed that the abovementioned pathways included many elements of the cytochrome P450 Cyp2 family (*Cyp2c54, Cyp2c38, Cyp2c40, Cyp2c29, Cyp2c50, Cyp2f2, Cyp2b10, Cyp2a1*) and glutathione metabolism genes (*Gsta3, Gsta4*).

### Transcriptomics validation by qPCR and by functional studies of cytochrome P450

As Cyp2c genes are associated with multiple essential liver functions, from drug metabolism to inflammatory mediators’ production^15^, we decided to validate those changes and evaluate their dynamics at 4-time points after burn injury. From the differentially expressed genes in Fig. 2, we selected representatives of the cytochrome P450 Cyp2c family, namely *Cyp2c29, Cyp2c38, Cyp2c40, and Cyp2c54*. We measured their expression in the 4 study groups at 4 different time points – 6 h, 9 h, 12 h, and 24 h after burn injury (Supplemental Fig. 1). We confirmed the downregulation of their expression at the 24 h time point after burn in both age groups. The transcriptomics validation of multiple P450 cytochrome genes in a time course study confirms that the downregulation is initially mild and becomes more severe as time progresses postburn (Supplementary Fig. 1).

### Common metabolomic response to burn injury in young and aged mice

**Fig. 3:**
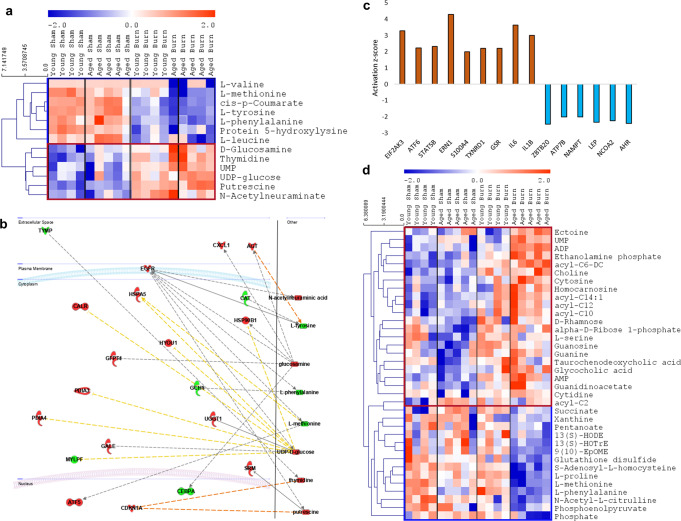
Metabolomic analysis of liver response to burn stimuli in aged and young mice. **a** A heatmap of differentially expressed metabolites in both aged burn versus aged sham and young burn mice versus young sham ones (*p* < 0.05 for each comparison). The blue cluster shows metabolites with a lower level in aged burn mice, and the red cluster shows metabolites with a higher level in aged burn mice. **b** Network of differentially expressed genes and metabolites in both young and aged mice after burn injury (only nodes having any connections were shown). **c** Upstream regulators of metabolic and transcriptomic changes after the burn injury are common for both aged and young mice. Only experimentally proven data for hepatocytes and/or liver was used for the prediction. **d** A heatmap of differentially expressed metabolites between aged burn and young burn mice (*p* < 0.05). In network, red and green colors mark up- and down-regulated nodes found in either metabolomic or transcriptomic data. *N* = 5 per group.

Cytochrome P450 genes are closely associated with many metabolic pathways^16^. Thus, we analyzed liver samples’ metabolite in young and aged mice. Metabolomic profiling of liver samples indicated that mice, irrespective of age, responded to burn injury by lowering their levels of many amino acids, including valine, methionine, tyrosine, phenylalanine, and leucine as well as cis-p-coumarate and protein 5-hydroxylysine. We also observed increased levels of glucosamine, UMP (uridine monophosphate), UDP-glucose (uridine diphosphate glucose), thymidine, putrescine, and N-acetylneuraminate (Fig. 3a, Supplementary Data 11).

Next, we connected the observed dysregulated metabolites with previously selected commonly differentially expressed genes in both aged and young mice after burn injury (Fig. 2). In our network analysis, we looked for a connection between two datasets via the Ingenuity Pathway Analysis tool. The created network showed many associations between differentially expressed metabolites and genes that were commonly dysregulated by burn injury in both aged and young mice (Fig. 3b). Interestingly, Egfr (epidermal growth factor receptor), a membrane receptor, was found to be highly associated with many disturbed metabolites suggesting its crucial role in the development of post-injury metabolomic disturbances^17,18^. Additionally, we looked for upstream regulators of all commonly observed changes in metabolites and genes (Fig. 3c). We found 9 upstream regulators predicted to be activated. Among them, we found two kinases: Eif2ak3 (Eukaryotic Translation Initiation Factor 2 Alpha Kinase 3), Ern1 (Endoplasmic Reticulum to Nucleus Signaling 1); two transcription regulators: Atf6 (Activating Transcription Factor 6), Stat5b (Signal Transducer And Activator Of Transcription 5B); two enzymes associated with glutathione metabolism: Txnrd1 (Thioredoxin Reductase 1), Gsr (Glutathione-Disulfide Reductase); two cytokines: IL1b, IL6, and one hypoxia-inducible gene: S100a4 (S100 calcium binding protein A4). Inhibition of six upstream regulators was also predicted: two transcription regulators: Zbtb20 (Zinc Finger and BTB Domain Containing 20), Ncoa2 (Nuclear Receptor Coactivator 2); one transporter (Atp7b - ATPase Copper Transporting Beta), one cytokine - visfatin (Nampt), one growth factor - leptin (Lep), and one ligand-dependent nuclear receptor (Ahr - Aryl Hydrocarbon Receptor). The molecular targets of those master regulators found to be dysregulated in our transcriptomic data are shown in Supplementary Data 12. Interestingly, inhibition of Zbtb20 could be responsible for observed Cyp2 downregulation (by targeting molecules like *Cyp1a2, Cyp2c54, Cyp2c8, Cyp2f1*) and inhibition of leptin (by targeting: *Cyp2c54, Cyp2c8, Cyp2f1, Cyp7a1*)^19^.

### Metabolomic differences in response to burn injury between young and aged mice

Having looked at common metabolomic changes after burn injury in both aged and young mice, next, we also looked for the differences. Firstly, we found that among some of the commonly burn-dysregulated metabolites, three had more pronounced changes in aged mice, including deeper downregulation of phenylalanine and methionine levels and higher up-regulation of UMP levels in aged *vs*. young mice (Fig. 3d). Additional disturbances, not present in young mice, were identified in aged burn mice, and included: downregulation of phosphate, S-adenosyl-L-homocysteine, L-proline, glutathione disulfide and 9(10)-EpOME (epoxyoctadecenoic acid); and up-regulation of guanosine, ADP (adenosine diphosphate), ethanolamine phosphate, acyl-C6-DC (methylglutarylcarnitine) and ectione.

### In silico drug predictions

**Fig. 4:**
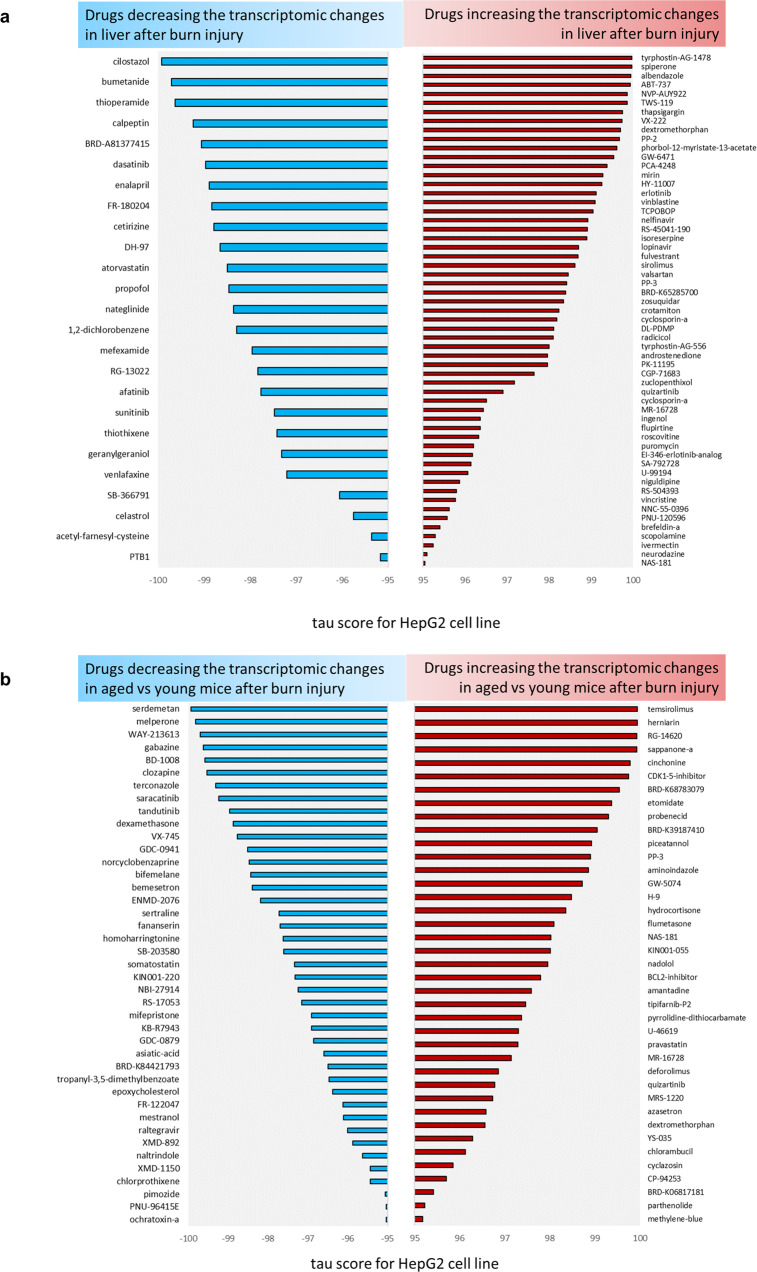
In silico drug predictions. **a** Predictions of drugs able to cause similar (right panel, red columns) or reverse effect (left panel, blue columns) in HepG2 cell line as common transcriptional changes observed after burn injury in the liver in both aged and young mice. **b** Predictions of drugs performed for the transcriptional difference observed after burn injury between aged and young mice. Only drugs with HepG2 tau score > |95| are shown.

Finally, we searched for drugs that could modify the liver burn injury response. Thus, we performed an in silico analysis of drugs that could cause similar or opposite effects to the transcriptomic differences observed in burn response among aged and young mice. To do so, we used the Connectivity map tool - Cmap. Providing the list of commonly dysregulated genes from Fig. 2, we predicted which drugs could cause a similar effect to burn injury (Fig. 4a right panel). The predicted drug list included commonly used drugs like albendazole, dextromethorphan, and valsartan. Assuming that observed expression changes after burn injury are harmful, the abovementioned drugs could exacerbate them. We also looked at drugs that have opposite expression effects. We found widely used drugs like enalapril, atorvastatin, and cilostazol. Those drugs could cause the opposite transcriptional effect, potentially reversing transcriptomic changes observed in mice liver after burn injury (Fig. 4a left panel).

Additionally, we searched for drugs able to cause similar and opposite expression changes to those observed between aged burn *vs*. young burn mice (Fig. 4b). Among drugs causing similar transcriptional effects to the one observed in aged *vs*. young mice after burn injury; we found such commonly used drugs as etomidate, probenecid, flumetazone and nadolol (Fig. 4b – right panel). Among drugs with opposite expressional effect to the one observed in aged burn mice (compared to young burn individuals) were clinically relevant drugs such as melperone, clozapine, dexamethasone, and sertraline (Fig. 4b – left panel).

## Discussion

Burns are a significant cause of morbidity and mortality worldwide, with an estimated 11 million cases yearly and more than 300,000 fatalities^20^. Burns affect seemingly every age group^2^. However, the mortality rate is significantly higher in elderly burn patients^21^. Regardless of age, severe burns cause damage to multiple organs distant from the original burn wound, leading to severe clinical problems such as multiple organ dysfunction syndrome (MODS)^3^. The liver is particularly affected after burn injury exhibiting hepatomegaly and fatty infiltration^5^. BILD is characterized by edema and cellular damage leading to leakage of hepatic enzymes AST and ALT into the circulation^4^. Clinical studies have demonstrated that the mortality in burn victims is elevated in patients with persistently elevated AST and ALT^8^. Advanced age is associated with cellular derangements in the liver^22^. Several age-related hepatic changes include a decline in liver volume, an increase in the hepatic dense body compartment (lipofuscin), a reduction in drug metabolism, and diminished hepatobiliary functions^21–23^. Based on these correlations, it is not unreasonable to suggest that elderly patients are more susceptible to liver damage after suffering burns. With its metabolic, inflammatory, and immune functions, the liver is fundamental for patient survival and recovery following burn injury^4^. Therefore, it is logical to study the liver and attempt to elucidate modifiable factors that specific targeted interventions could improve the outcomes of burn victims.

We evaluated the liver omics at a base level, comparing young and aged sham animals. In this study, the transcriptomics analysis has demonstrated that while the livers from aged sham animals exhibit higher immunoglobulin gene expression and increased activity of IL-17 signaling pathways, they do not present significant downregulation of genes or signaling pathways compared to the livers of younger counterparts. These results match common age-related findings contributing to the characteristic chronic inflammatory state of aged individuals known as inflammaging^24^.

Following a significant trauma such as a burn, the liver physiology drastically redirects its protein synthesis favoring the production of acute phase reactants^5^. As the liver becomes hyper-functional in producing these essential proteins to survive a burn insult, it reduces the production of constitutional proteins, steroid hormones, bile, and numerous cytochrome p450 metabolic processes^4,25^. As such, in this study, the transcriptomics analysis showed that after burn, the foremost up-regulated pathways in the livers of young and aged mice involved protein processing in the endoplasmic reticulum, protein export, fructose, and mannose metabolism, and IL-17 signaling. The major hepatic pathways downregulated after a burn in young and aged mice were chemical carcinogenesis, steroid hormone biosynthesis, and metabolism of xenobiotics by cytochrome P450.

Additionally, our data demonstrate that after burn, the livers of aged differently from younger mice profoundly downregulate processes such as steroid hormone biosynthesis, bile secretion, linoleic acid, arachidonic acid, and retinol metabolism, arginine biosynthesis, and metabolism of xenobiotics by cytochrome P450. While prioritization of acute phase proteins benefits survival rate at an initial phase post-injury, a severe and prolonged downregulation of other hepatic functions, such as cytochrome metabolism, may be detrimental for burn victims, especially in the elderly^26^. There is a lack of understanding about the impact of these processes on the outcomes in young and aged burn victims, which can be an important focus of future research.

Particularly distinctive was the downregulation of cytochrome P450 Cyp2 family genes involved in multiple liver functions, including protein and lipid metabolism, oxidative stress buffers, and metabolism of drugs and toxins^27^. These differences can result in a toxin metabolism shift in the liver cells and destabilization of free radical scavenging and DNA replication, recombination, and repair processes in aged animals after burn injury^28,29^. The cytochrome P450 system may also be affected by inflammation and age^30^. Two cytochrome P450 enzyme families, Cyp2c and Cyp2j, are responsible for metabolizing proinflammatory metabolites (arachidonic acid) to anti-inflammatory compounds (epoxyeicosatrienoic acids)^31^. In the data presented herein, all 11 members of the Cyp2c family identified in our liver samples (Cyp2c54, Cyp2c38, Cyp2c40, Cyp2c29, Cyp2c50, Cyp2c68, Cyp2c70, Cyp2c39, Cyp2c67, Cyp2c37, Cyp2c69) and one detected CYP2J member - Cyp2j5 - were found to be downregulated among liver of aged burn mice compared to the young burn mice.

Clinical evidence reveals that older patients have an attenuated inflammatory response to burn injury relative to younger injured subjects, followed by a hyper-inflammatory reaction^21^. Microsomal oxidation deceleration driven by cytochrome P450 was shown to play a critical role in developing MODS due partly to the accumulation of endogenous toxic compounds^27^. MODS was found to have increased incidence in the elderly after burn injury, but this was not accompanied by increased infection or sepsis rate^21^. Moreover, decreased cytochrome P450 Cyp2c activity could accumulate toxic metabolites from burn treatment medications as these cytochrome family members are mainly involved with drug metabolism^32^. Those observations suggest that inhibiting cytochrome P450 Cyp2 as a component of the hepatic post-burn inflammatory response may have detrimental effects and that increased expression of those enzymes could have a potentially beneficial effect. However, further studies need to establish whether the downregulation of Cyp2c after burn injury is responsible for less favorable clinical outcomes.

Given that the cytochrome p450 system is abundant in the liver playing the role of numerous functions^33^, we validated our transcriptomics findings by measuring the gene expression of central genes that operate in this system in 2 different mice strains (Balb/c and C57BL/6). In a time-course study, the transcriptomics validation of multiple P450 cytochrome genes demonstrates that the downregulation becomes more severe as time advances postburn. These findings raise a red flag in treating burn patients, indicating that drug metabolism may be severely affected in the first weeks after burns.

The metabolomic analysis exhibited a common and a different hepatic response pattern to burns between young and aged animals. The common response showed a decrease in amino acid levels, including valine, methionine, tyrosine, phenylalanine, leucine, cis-p-coumarate, and protein 5-hydroxylysine. Most of these metabolites are essential amino acids, and their decrease is commonly associated with a lack of nutritional intake^34^. Postburn metabolism not only consumes essential amino acids but also decreases appetite, which can result in an essential amino acid deficiency^35^. Liver injury can cause a decrease in several catabolic pathways resulting in the accumulation of several metabolites^36^. In the liver of mice postburn, we observed an increased level of glucosamine, UMP (uridine monophosphate), UDP-glucose (uridine diphosphate glucose), thymidine, putrescine, and N-acetylneuraminic, indicating that energy and proliferation catabolic pathways are affected in this pathology (Fig. 4a, Supplementary Data 11).

We used an Ingenuity Pathway Analysis tool to further evaluate the common hepatic response to burns to create a network connecting the metabolomic and transcriptomics changes among the four study groups. The network showed many connections between differentially expressed metabolites and genes that were commonly dysregulated by burn injury in both aged and young mice (Fig. 3b). Egfr was found to be highly associated with many disturbed metabolites suggesting its crucial role in the development of post-injury metabolomic disturbances. EGFR is pivotal in hepatocyte proliferation, liver regeneration, liver steatosis, oxidative stress, mitochondria dysfunction, and cell death in different animal models of liver injury^18^. EGFR activation is achieved ligand-based or ligand-independent, and the latter is mainly associated with liver injury. Given EGFR’s diverse involvement at many levels in hepatic pathophysiological processes, additional research is necessary to determine if EGFR may be used as a therapeutic target to treat liver disorders, including burn induce liver damage^17^. The IPA analysis of the common hepatic response to burn yield 6 deactivated master regulators: Zbtb20, Ncoa2, Atp7b, visfatin (Nampt), leptin (Lep), and Aryl Hydrocarbon Receptor (Ahr). Interestingly, inhibition of Zbtb20 could be responsible for observed Cyp2 downregulation (by targeting molecules like Cyp1a2, Cyp2c54, Cyp2c8, Cyp2f1) and inhibition of leptin (by targeting: Cyp2c54, Cyp2c8, Cyp2f1, Cyp7a1)^19^.

The significant differences in metabolic pathways noticed in the livers of young burn and aged burn mice involved a severe downregulation of glutathione metabolism and citrate cycle (TCA cycle) in the aged animal group. These metabolomics results match our previous findings that highlight the presence of a disrupted antioxidant glutathione system in the liver of aged animals, which can lead to increased susceptibility to hepatic damage following burns^10^.

In silico (computer-based) drug prediction and repurposing have become one of the most used methods to make drug discovery more accurate and economical^37^. This method seeks to recognize the relationship network between the target and the drug, which is attained through bioinformatics tools and public databases^38^. In this study, we used a Connectivity map tool – Cmap, to obtain drugs that can induce opposite and similar transcriptomics and metabolomics changes in hepatocytes as the ones caused by burn injury in our experiments. We hypothesize that harnessing side targets of clinically used drugs might help reprogram liver cells to handle better the stress of responding to a burn injury.

Some predicted drugs, like atorvastatin, enalapril, cetirizine, cilostazol, and venlafaxine, are widely used in clinical practice. Based on their mechanism of action, these drugs should not affect the immune system’s response to burn injury, as anti-inflammatory drugs are used to ameliorate organ damage, such as dexamethasone. They might be considered an additional treatment for elderly patients after burn injury. Interestingly, histamine receptor antagonist was found previously to accelerate skin barrier repair. Thus, using cetirizine could have another beneficial effect in treating burns^39^.

The list of drugs our in silico search identifies involves several FDA-approved compounds used to treat common conditions. Drugs that induce “similar changes” could harm the organ and may be avoided when treating burn injury patients. Drugs that cause “opposite changes” should be further evaluated in animal models of burn-induced liver injury to validate the calculated effect predicted by our analysis.

Our approach via the expression data analysis by Cmap was previously used to predict targeted combination treatment for pancreatic cancer successfully^40,41^. Thus, we expect that some of the predicted drugs will prove to be beneficial for burn victims. However, the recommendation of using any of the predicted medications in a clinical scenario needs to be supported by in-depth in vivo drug studies and clinical trials as our results have a preliminary character. More follow-up studies are needed to validate the concept and develop a new treatment for burn injury patients.

We accept the murine model’s shortcomings and recognize that these models do not always replicate clinical observations. The animals are genetically similar and live in a controlled environment. While burn patients are treated in a burn intensive care unit (ICU), which provides continuous intravenous fluids, antibiotics, nutrition, and monitoring, the absence of a rodent ICU creates a drastically different treatment environment, adding extra variables that can affect the outcomes of experiments. Rodent skin has a different structure and physiology than human skin, and the quantity and quality of the inflammatory mediators produced by the damaged skin may differ from human skin. Environmental factors originate from many vendors of mice and may have an impact on the outcomes of the research.

In this investigation, the weight difference between young and old mice was around 3–5 grams, which might affect the findings. This study was performed using female mice, given that publications demonstrate higher mortality rates in women who sustain burn injuries than men; however, sex differences can impact the results in murine models of burn injuries^42–44^.

Since this study used the whole liver for transcriptomics and metabolomics analysis, we recognize that other parenchymal and immune cells in the liver can greatly impact the liver transcriptome/metabolome, which should be considered when analyzing this data. However, other studies showed that one day after skin burn injury, there is no significant increase of neutrophils infiltration MPO activity in liver samples^45^.

In conclusion, we analyzed the gene expression and metabolic responses to burn injury in the livers of aged and young mice and found multiple similarities and differences between them. A significant difference in the livers of aged animals after a burn is a profound downregulation of essential enzymatic superfamily such as the cytochrome P450. By using in silico analysis, we predicted commonly used drugs that may rewire the burn-induced liver response to a more favorable one and thus increase the survival of burn victims. Further study is warranted to evaluate the effect of these drugs on alleviating the burn injury in the aged mice.

## Methods

### Mice

Young 3–4 months old (equivalent to 20–25 human years) BALB/c and C57BL/6 female mice were obtained from The Jackson Laboratory (Bar Harbor, ME). Aged 20–22 months old (equivalent to 65–70 human years) BALB/c and C57BL/6 female mice from the National Institute of Aging (NIA) Colony (Charles River Laboratories, Wilmington, MA). All omics experiments were performed on BALB/c mice, while both strains were used for qPCR validation. Before the experiments, all mice were housed at the University of Colorado Anschutz Campus’s vivarium for two weeks. Mice were maintained on a 12-hour light/dark cycle with lights on at 8:00 am. All animals were kept in cages with controlled temperature (23 °C–24 °C) and humidity (60% ± 10%). The mice were fed with certified standard chow and tap water ad libitum. The experiments were performed between 9 and 11 am to decrease variability caused by differences in circadian rhythms. All procedures were approved by The University of Colorado Institutional Animal Care and Use Committee.

The ARRIVE guidelines were used to ensure proper reporting of methods, results, and discussion.

### Burn injury model

We distributed mice randomly into four experimental groups (young sham, young burn, aged sham, and aged burn). To create a controlled burn injury, we used the method described by Faunce et al.^46^. The mice were anesthetized with isoflurane, 55.5 mg/kg of ketamine Intra peritoneal (lP), and 2.6 mg/kg of xylazine IP (Webster Veterinary, Sterling, MA). While anesthetized, each mouse’s dorsum was shaved. Each mouse was then placed individually in a plastic template designed to expose 15% total body surface area (TBSA) of skin calculated as described^47^. Mice were then exposed to 94 degrees Celsius (for the burn groups) or room temperature water baths (for the sham groups) for 8 s as previously described^46,48^. Following this method, exposure of the mice’s dorsum to 94 degrees Celsius water induces a localized full-thickness skin burn^46^. After this procedure, each mouse received additional pain medication (0.13 mg/kg buprenorphine IP) and normal saline IP for resuscitative purposes according to the parkland formula (4 × body weight (kg) × TBSA(%)). The mice were assessed and monitored through the post-procedure recovery preserving normothermia using warm pads. Lastly, the mice were humanly euthanized using CO_2_ followed by cervical dislocation, 24 h (for transcriptomics and metabolomics data) and at multiple points 6-, 9-,12-, and 24 h in a time course experiment (for transcriptomics validation) after the burn injury procedure.

### Tissue and sample processing

Following euthanasia, the livers of all mice were infused with ice-chilled saline via the portal vein. For consistency, the right hepatic lobe in each animal was snap frozen in liquid nitrogen and then stored at −80 °C for further analysis. Livers were maintained in tubes on dry ice when pieces were removed for homogenization, and homogenized tissue was kept in tubes on ice prior to analysis.

### RNA preparation and gene expression analysis

RNA was extracted from liver using a RNeasy mini kit (Qiagen, Hilden, Germany) as previously described^9–11^, following the manufacturer’s recommended protocol. cDNA was then synthesized using an iScript kit according to manufacturer protocol (Bio-Rad, Hercules, CA). Quantitative RT-PCR was was performed using Universal SYBR Green Supermix (BioRad, #1725124). Standard desalted primers were purchased from Sigma. *Cyp2c29* Forward Primer: 5′ GCTCTCCTACTCCTGCTGAAGT 3′ Reverse Primer: 5′ ATGTGGCTCCTGTCTTGCATGC 3′ *Cyp2c38* Forward Primer: 5′ CCTTGTCCCTAACAACCTACCC 3′ Reverse Primer: 5′ GGAACTCCTTGCTGTCATGCAG 3′ *Cyp2c40* Forward Primer: 5′ CAAGAGGAAGCACAGTGGCTCA 3′ Reverse Primer: 5′ GGAAAACAATGGAGCAGATGACAT 3′ *Cyp2c54* Forward Primer: 5′ GGACATCTGCCAATCCTTCACC 3′ Reverse Primer: 5′ GGTCAACCAGAGCTTCCTTCAC 3′ run on a QuantStudio 3 Real-Time PCR System (Applied Biosystems, Foster City, CA). Gene expression was quantified using the ΔΔCt algorithm, with Gapdh as the endogenous control (Cat No. 4352339E, Applied Biosystems, Foster City, CA).

### RNA sequencing

The Genomics Shared Resource performed RNA sequencing in the Cancer Center shared resource core at the University of Colorado Anschutz Medical Campus. 100 ng of total RNA was used to construct an Illumina-compatible mRNA library using Tecan Universal Plus mRNA-SEQ kit Cat No. 0520-A01. Sequencing was done on an Illumina NovaSEQ6000 Instrument using an S4 Flow Cell paired-end sequencing 2 × 150. FASTQ files were delivered after standard demultiplexing procedures were used, converting.bcl files to.seq files using CASAVA software as described^49,50^.

### Metabolomic analysis

The Mass Spectrometry Metabolomics Shared Resource Facility at the University of Colorado Anschutz Medical Campus processed and analyzed the metabolomics data. Briefly, mass spectrometry-based metabolomics was performed on frozen liver samples. For polar metabolites, tissues were extracted at 15 mg per mL in ice-cold lysis/extraction buffer (methanol:acetonitrile:water 5:3:2), and 10 µL of supernatants were analyzed using a 5 min C18 gradient in positive and negative ion modes (separate runs)^51,52^. For polar lipids, tissues were extracted at 7.5 mg per mL in cold methanol containing stable isotope-labeled standards (see attached list), and 10 µL of supernatants were analyzed using a 17 min gradient in negative ion mode as previously described^53^. Data was acquired on a Thermo Vanquish UHPLC system coupled online to a Thermo Q Exactive mass spectrometer. Data analysis was performed in Maven against an in-house compound library as described^52,53^.

### Statistics and data reproducibility

The NetworkAnalyst platform was used to perform differential expression analysis with EdgerR test after filtering out genes with variance lower than 15 and abundance lower than 4 and transforming data with upper quantile normalization (Supplementary Data 1–4). Data visualization for transcriptomic analysis was performed with volcano plots and principal component analysis^54^. For the transcriptomics analysis, statistical significance was considered with adj. *p* < 0.05 values with *n* = 3 per group. For transcriptomics validation, qPCR data were analyzed in 100 mice (20 Balb/c and 80 C57BL/6) at different time points (6-,9-12-, and 24 h after burn; *n* = 5 per study group). We used Graph Pad Prism 9.3.1. The D’Agostino-Pearson normality test was used to check the normal distribution of the variables. For the statistical analysis of variables with a normal distribution, the student *t*-test or ANOVA was used, whereas, for variables with a non-normal distribution, the Mann–Whitney *U* test was used. A *p* value < 0.05 was considered significant, and all tests were two-sided.

Heatmap from metabolomic data was performed with Multiple Experiment Viewer (MeV 4.8)^55^; a *p* value lower than 0.05 was considered statistically significant with *n* = 5 per group. Overrepresentation analysis was performed with the g:Profiler^56^ using the KEGG database.

The experimental design was presented with graphics designed with the BioRender platform.

### In silico analysis

To perform a functional analysis, construct networks, and predict upstream regulators, we used QIAGEN’s Ingenuity® Pathway Analysis (IPA®, QIAGEN Redwood City, www.qiagen.com/ingenuity). Network of direct and indirect connections of differentially expressed genes and metabolites overlapping for both young and aged mice after burn injury were used to feed the IPA algorithm. Nodes without any connections were excluded from the analysis. Prediction of upstream regulators of metabolic and transcriptomic changes after the burn injury was performed only using experimentally proven data for hepatocytes and/or liver from Qiagen knowledge base.

In silico drug repurposing prediction was done using the Connectivity map platform (https://clue.io/)^57^. The platform correlates the observed gene set dysregulation with known effects of perturbations on multiple cell lines. We narrowed our analysis to drugs tested on HepG2 cell lines as the closest to our experimental setting. For our analysis threshold for tau score >95 was considered to select drugs with similarity signature to our query, and <−95 for selecting drugs with the opposing signature. Tau score is a standardized measure ranging from −100 to 100, corresponding to the fraction of reference gene sets with significant similarity to the drug than the current query.

### Reporting summary

Further information on research design is available in the Nature Portfolio Reporting Summary linked to this article.

## Supplementary information

Idrovo_Peer Review File

Supplementary Information

Description of Additional Supplementary Files

Supplementary Data 1–12

Reporting Summary

## Data Availability

All data produced within this manuscript were attached as ‘Supplementary Materials’ file. Raw sequencing data are available in Sequence Read Archive (SRA) under following number: SUB13385022.
