# Supplementary material for: Transcriptomics, metabolomics, and in-silico drug predictions for liver damage in young and aged burn victims
Source: Commun Biol. 2023 Jun 2;6:597. doi: 10.1038/s42003-023-04964-2 (PMC10238406; doi:10.1038/s42003-023-04964-2)
Supplement: Supplementary file 2 — Supplementary Information [file 42003_2023_4964_MOESM2_ESM.pdf]

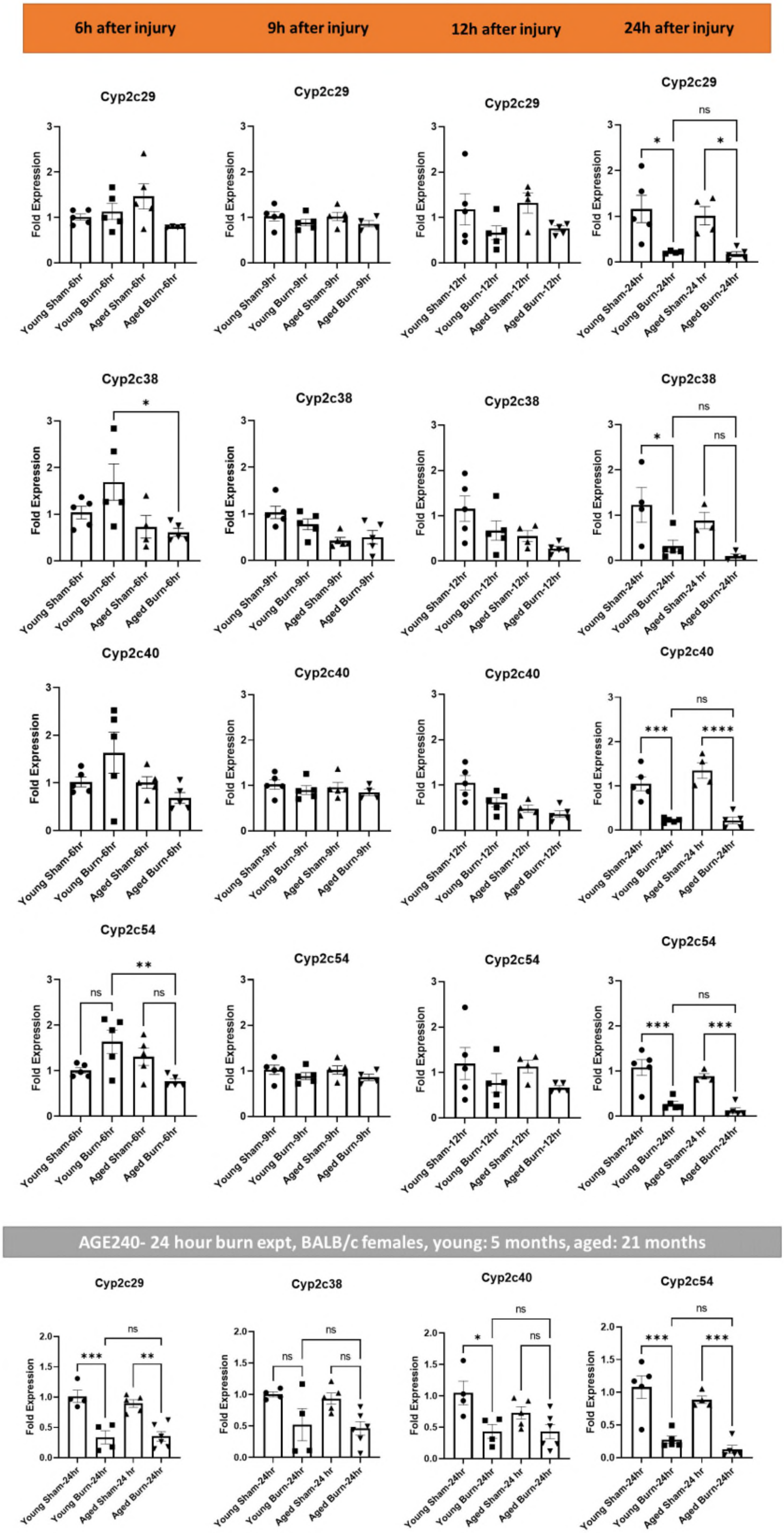

Supplementary Figure 1 – qPCR expression measurements in mice liver of 4 cytochrome P450 Cyp2c family members – Cyp2c29, Cyp2c38. Cyp2c40, Cyp2c54 - in four time points (6h, 9h, 12h, 24h) after the burn injury. Mean and standard error of mean were shown. N=5 per group.
