# Supplementary material for: Transcriptomics, metabolomics, and in-silico drug predictions for liver damage in young and aged burn victims
Source: Commun Biol. 2023 Jun 2;6:597. doi: 10.1038/s42003-023-04964-2 (PMC10238406; doi:10.1038/s42003-023-04964-2)
Supplement: Supplementary file 3 — Description of Additional Supplementary Files [file 42003_2023_4964_MOESM3_ESM.pdf]

Description of Additional Supplementary Files

File name: Supplementary Data 1

Description: EdgeR analysis – Differential expression analysis of liver transcriptomics data of aged burn and aged sham groups.

File name: Supplementary Data 2

Description: EdgeR analysis – Differential expression analysis of liver transcriptomics data of young burn and young sham groups.

File name: Supplementary Data 3

Description: EdgeR analysis – Differential expression analysis of liver transcriptomics data of aged burn and young burn groups.

File name: Supplementary Data 4

Description: EdgeR analysis – Differential expression analysis of liver transcriptomics data of aged sham and young sham groups.

File name: Supplementary Data 5

Description: Over-representation analysis results – KEGG pathway overrepresentation analysis results for genes up-regulated by burn injury in both aged and young mice in comparison to sham conditions.

File name: Supplementary Data 6

Description: Over-representation analysis results – KEGG pathway overrepresentation analysis results for genes down-regulated by burn injury in both aged and young mice in comparison to sham conditions.

File name: Supplementary Data 7

Description: Over-representation analysis results – KEGG pathway overrepresentation analysis results for genes up-regulated in aged sham group in comparison to young sham group.

File name: Supplementary Data 8

Description: Over-representation analysis results – KEGG pathway overrepresentation analysis results for genes down-regulated in aged sham group in comparison to young sham group.

File name: Supplementary Data 9

Description: Over-representation analysis results – KEGG pathway overrepresentation analysis results for genes up-regulated in aged burn group in comparison to young burn group.

File name: Supplementary Data 10

Description: Over-representation analysis results – KEGG pathway overrepresentation analysis results for genes down-regulated in aged burn group in comparison to young burn group.

File name: Supplementary Data 11

Description: Liver metabolomics analysis of aged burn, aged sham, young burn, and young sham mice 24 after injury.

File name: Supplementary Data 12

Description: Ingenuity Pathway Core Analysis results of genes and metabolites commonly dysregulated after the burn injury in both aged and young mice. Upstream regulators with predicted activation or inhibition were shown.
