# Supplementary material for: Transcriptomics, metabolomics, and in-silico drug predictions for liver damage in young and aged burn victims
Source: Commun Biol. 2023 Jun 2;6:597. doi: 10.1038/s42003-023-04964-2 (PMC10238406; doi:10.1038/s42003-023-04964-2)
Supplement: Supplementary file 5 — Reporting Summary [file 42003_2023_4964_MOESM5_ESM.pdf]

nature portfolio

Corresponding author(s): Juan-Pablo Idrovo

Last updated by author(s): May 15, 2023

Reporting Summary

Nature Portfolio wishes to improve the reproducibility of the work that we publish. This form provides structure for consistency and transparency in reporting. For further information on Nature Portfolio policies, see our Editorial Policies and the Editorial Policy Checklist.

Statistics

For all statistical analyses, confirm that the following items are present in the figure legend, table legend, main text, or Methods section.

n/a	Confirmed	
☐	☒	The exact sample size (*n*) for each experimental group/condition, given as a discrete number and unit of measurement
☐	☒	A statement on whether measurements were taken from distinct samples or whether the same sample was measured repeatedly
☐	☒	The statistical test(s) used AND whether they are one- or two-sided<br>*Only common tests should be described solely by name; describe more complex techniques in the Methods section.*
☒	☐	A description of all covariates tested
☐	☒	A description of any assumptions or corrections, such as tests of normality and adjustment for multiple comparisons
☐	☒	A full description of the statistical parameters including central tendency (e.g. means) or other basic estimates (e.g. regression coefficient) AND variation (e.g. standard deviation) or associated estimates of uncertainty (e.g. confidence intervals)
☒	☐	For null hypothesis testing, the test statistic (e.g. *F*, *t*, *r*) with confidence intervals, effect sizes, degrees of freedom and *P* value noted<br>*Give P values as exact values whenever suitable.*
☒	☐	For Bayesian analysis, information on the choice of priors and Markov chain Monte Carlo settings
☒	☐	For hierarchical and complex designs, identification of the appropriate level for tests and full reporting of outcomes
☒	☐	Estimates of effect sizes (e.g. Cohen's *d*, Pearson's *r*), indicating how they were calculated

Our web collection on statistics for biologists contains articles on many of the points above.

Software and code

Policy information about availability of computer code

Data collection	FASTQ files were delivered after standard demultiplexing procedures were used, converting .bcl files to .seq files using CASAVA software as described.<br>Data was acquired on a Thermo Vanquish UHPLC system coupled online to a Thermo Q Exactive mass spectrometer. Data analysis was performed in Maven against an in-house compound library as described.
Data analysis	The NetworkAnalyst platform was used to perform differential expression analysis with EdgerR test after filtering out genes with variance lower than 15 and abundance lower than 4 and transforming data with upper quantile normalization (Table S1-4). Data visualization for transcriptomic analysis was performed with volcano plots and principal component analysis 22. For the transcriptomics analysis, statistical significance was considered with adj. p<0.05 values with n=3 per group. For transcriptomics validation, qPCR data were analyzed in 100 mice (20 Balb/c and 80 C57BL/6) in different time points (6-,9-12-, and 24 hours after burn) n= 5 per study group. We used Graph Pad Prism 9.3.1. The D'Agostino-Pearson normality test was used to check the normal distribution of the variables. For the statistical analysis of variables with a normal distribution, the student t-test or ANOVA was used, whereas, for variables with a non-normal distribution, the Mann–Whitney U test was used. A p-value <0.05 was considered significant, and all tests were two-sided.<br>Heatmap from metabolomic data was performed with Multiple Experiment Viewer (MeV 4.8) 23; a p-value lower than 0.05 was considered statistically significant with n= 5 per group. Overrepresentation analysis was performed with the g:Profiler 24 using the KEGG database.<br>The experimental design was presented with graphics designed with the BioRender platform.<br>In silico analysis<br>To perform a functional analysis, construct networks, and predict upstream regulators, we used QIAGEN's Ingenuity® Pathway Analysis (IPA®, QIAGEN Redwood City, www.qiagen.com/ingenuity). Network of direct and indirect connections of differentially expressed genes and metabolites overlapping for both young and aged mice after burn injury were used to feed the IPA algorithm. Nodes without any connections were excluded from the analysis. Prediction of upstream regulators of metabolic and transcriptomic changes after the burn injury was

performed only using experimentally proven data for hepatocytes and/or liver from Qiagen knowledge base.
In silico drug repurposing prediction was done using the Connectivity map platform (https://clue.io/) 25. The platform correlates the observed gene set dysregulation with known effects of perturbations on multiple cell lines. We narrowed our analysis to drugs tested on HepG2 cell lines as the closest to our experimental setting. For our analysis threshold for tau score >95 was considered to select drugs with similarity signature to our query, and <-95 for selecting drugs with the opposing signature. Tau score is a standardized measure ranging from -100 to 100, corresponding to the fraction of reference gene sets with significant similarity to the drug than the current query.

For manuscripts utilizing custom algorithms or software that are central to the research but not yet described in published literature, software must be made available to editors and reviewers. We strongly encourage code deposition in a community repository (e.g. GitHub). See the Nature Portfolio guidelines for submitting code & software for further information.

Data

Policy information about availability of data

All manuscripts must include a data availability statement. This statement should provide the following information, where applicable:

- Accession codes, unique identifiers, or web links for publicly available datasets
- A description of any restrictions on data availability
- For clinical datasets or third party data, please ensure that the statement adheres to our policy

We provided all data as Supplementary files to the manuscript.

Human research participants

Policy information about studies involving human research participants and Sex and Gender in Research.

Reporting on sex and gender	NA
Population characteristics	NA
Recruitment	NA
Ethics oversight	NA

Note that full information on the approval of the study protocol must also be provided in the manuscript.

Field-specific reporting

Please select the one below that is the best fit for your research. If you are not sure, read the appropriate sections before making your selection.

☒ Life sciences ☐ Behavioural & social sciences ☐ Ecological, evolutionary & environmental sciences

For a reference copy of the document with all sections, see nature.com/documents/nr-reporting-summary-flat.pdf

Life sciences study design

All studies must disclose on these points even when the disclosure is negative.

Sample size	For RNA-seq experiemtn N=3 was chosen as commonly used number in high-through put experiment allowing for SD evaluation. For metabolomics data, due to higher expected variance, N=4 per groups was chosen. For validation experiments N=5 per group was chosen as standard protocol for animal studies.
Data exclusions	NA
Replication	One of the main findings, downregulation of Cyp2c gene family was validated by qPCR measurements.
Randomization	We distributed mice randomly into four experimental groups (young sham, young burn, aged sham, and aged burn).
Blinding	NA, no measurements were done in subjective manner so no blinding was necessary. Additionally, sham and skin burn injury interventions are visible, so it was not possible to hide group allocation from the experimentators.

Reporting for specific materials, systems and methods

We require information from authors about some types of materials, experimental systems and methods used in many studies. Here, indicate whether each material, system or method listed is relevant to your study. If you are not sure if a list item applies to your research, read the appropriate section before selecting a response.

Materials & experimental systems

n/a	Involved in the study
x	Antibodies
x	Eukaryotic cell lines
x	Palaeontology and archaeology
	x Animals and other organisms
x	Clinical data
x	Dual use research of concern

Methods

n/a	Involved in the study
x	ChIP-seq
x	Flow cytometry
x	MRI-based neuroimaging

Animals and other research organisms

Policy information about studies involving animals; ARRIVE guidelines recommended for reporting animal research, and Sex and Gender in Research

Laboratory animals	Young 3-4 months old (equivalent to 20-25 human years) BALB/c and C57BL/6 female mice were obtained from The Jackson Laboratory (Bar Harbor, ME). Aged 20-22 months old (equivalent to 65-70 human years) BALB/c and C57BL/6 female mice from the National Institute of Aging (NIA) Colony (Charles River Laboratories, Wilmington, Mass). Before the experiments, all mice were housed at the University of Colorado Anschutz Campus's vivarium for two weeks.
Wild animals	NA
Reporting on sex	Female mice were used across whole study.
Field-collected samples	NA
Ethics oversight	All procedures were approved by The University of Colorado Institutional Animal Care and Use Committee.

Note that full information on the approval of the study protocol must also be provided in the manuscript.
