# Supplementary material for: Transcriptomics, metabolomics, and in-silico drug predictions for liver damage in young and aged burn victims
Source: Commun Biol. 2023 Jun 2;6:597. doi: 10.1038/s42003-023-04964-2 (PMC10238406; doi:10.1038/s42003-023-04964-2)
Supplement: Supplementary file 1 — Idrovo_Peer Review File [file 42003_2023_4964_MOESM1_ESM.pdf]

nature portfolio

Peer Review File

Transcriptomics, metabolomics, and in-silico drug predictions for liver damage in young and aged skin-burn victims

Open Access This file is licensed under a Creative Commons Attribution 4.0 International License, which permits use, sharing, adaptation, distribution and reproduction in any medium or format, as long as you give appropriate credit to the original author(s) and the source, provide a link to the Creative Commons license, and indicate if changes were made. In the cases where the authors are anonymous, such as is the case for the reports of anonymous peer reviewers, author attribution should be to 'Anonymous Referee' followed by a clear attribution to the source work. The images or other third party material in this file are included in the article's Creative Commons license, unless indicated otherwise in a credit line to the material. If material is not included in the article's Creative Commons license and your intended use is not permitted by statutory regulation or exceeds the permitted use, you will need to obtain permission directly from the copyright holder. To view a copy of this license, visit http://creativecommons.org/licenses/by/4.0/.

Reviewers' comments:

Reviewer #1 (Remarks to the Author):

The study is to investigate the mechanism of acute post-burn liver response in aged mice. By comparing 20-22 months-old and 3-4 months-old mice, they observed the same and different liver responses in transcriptional and metabolic levels. The transcriptomics data Cyp2c genes were further validated by qRCR. Lastly in silico drug prediction was performed for the future therapeutic potentials of liver treatment. The study has a great impact on clinical concerns and the novelty of in silico screening. The experiment design and data report are well organized. The detailed results such as specific genes and drugs revealed the directions in the future study. The conclusion was supported by the evidence report.
Reviewer congratulated the authors' great work and listed comments below:
• Line 92, 3-4 months and 20-22 months old mice were enrolled in the study. Aged mouse has different body weight and body size from the young. Liver response depends on burn severity (% tbsa). Though the current burn model has been well established over the years, authors could give more detail information (i.e, burn molds) to describe the model better in the study.
• Line 94, The animal model has been well established following Dr. Plackett Studies since 2003, which initially studied immunity response after burn injury. Are there any rationales of choosing two strains of mice to conduct the validation in this study? Also, the model was based on female mouse. Would authors comment whether the gender difference affects the data interpretation in the burn animal model?
• Line 98, the condition of food intake with food component could be added since the study explores the metabolic changes in mice.
• Line 103, please check the typo "so."
• Line 109, 1ml of saline was applied IP on burned mice. Have authors considered the different amount of resuscitation fluid required in young and aged mice after receiving 15% tbas burn? Does the burn animal receive the sufficient saline for resuscitation?
• Line 112, please describe euthanasia method.
• Line 119, please provide RNA extraction/quality method, and qPCR method.
• Line 142, qPCR validation were taken 160 mice total in the study. please justify the rationale of the animal amount based on the animal experiment 3Rs principle.
• Line 363, please clarify if "b57bl" is a typo.

Reviewer #2 (Remarks to the Author):

In this manuscript, Malachowska et al., analyzed transcriptomics and metabolomics data from the liver of young and aged mice suffering burn-induced trauma. The study highlights pathway interactions and master regulators that underlie the liver response to burn injury in young and aged mice. Also, computer-based in-silico analysis was conducted to predict therapeutic targets to prevent or reverse burn-induced liver damage.
Although the reviewer agrees with the authors that the work will add some significant knowledge to the understanding of the pathophysiology of burn-induced trauma from the perspective of the liver, the study is in some aspects somewhat preliminary. In particular, the conclusions for the therapeutic targets to prevent or reverse burn-induced liver damage is somewhat questionable. The following issues should be addressed prior publication.

Major issue:
1. Young and old BALB/c and C57BL/6 female mice were used in the study. Why were only female

mice were used? The authors should at least explain the reasons in the Method section.
2. The immune responses of BALB/c mice and C57BL/6 mice are very different. Why were two mice strains used and were these equally distributed between the groups?
Can the authors provide some relevant evidence that these two mice respond differently or similarly to burn-induced injury at the transcriptomic and metabolomic level in liver? Were there any strain differences noted in the analyses performed?
3. During trauma, large quantities of immune cells accumulate in the liver. Some of the upregulated genes found in this study are also marker genes of myeloid cells (such as S100a9, S100a8). The changes observed by the authors might not be restricted to responses in hepatocytes, and might be due to the massive recruitment of immune cells, particularly myeloid cells. Was histology performed to evaluate for leukocyte infiltration differences?
4. Description of liver sample collection require more information. Many details are missing, such as, the method of perfusion; red blood cells removal process before RNA extraction.
5. How was liver sampling standardized?
6. Supplemental figures were not uploaded and accessible.
7. The authors carry out a limited attempt at validation by running qPCR for Cyp2c. To markedly enhance the value of their screening study, they should determine if cytochrome p450 function is impaired in older mice after burn injury. This would validate the value of the screening approach.
8. Methods of the ‘in silico’ analysis and other analysis is needed.

Minor issue:
1. In Figure 1B and figure legends, the number of mice in each group is not noted or described.
2. Insertion of references is not standardized.

Reviewers' comments:

Reviewer #1 (Remarks to the Author):

The study is to investigate the mechanism of acute post-burn liver response in aged mice. By comparing 20-22 months-old and 3-4 months-old mice, they observed the same and different liver responses in transcriptional and metabolic levels. The transcriptomics data Cyp2c genes were further validated by qRCR. Lastly in silico drug prediction was performed for the future therapeutic potentials of liver treatment. The study has a great impact on clinical concerns and the novelty of in silico screening. The experiment design and data report are well organized. The detailed results such as specific genes and drugs revealed the directions in the future study. The conclusion was supported by the evidence report. Reviewer congratulated the authors' great work and listed comments below:
1. Line 92, 3-4 months and 20-22 months old mice were enrolled in the study. Aged mouse has different body weight and body size from the young. Liver response depends on burn severity (% tbsa). Though the current burn model has been well established over the years, authors could give more detail information (i.e, burn molds) to describe the model better in the study.

Author's response: We modified the following paragraph to add some details of the methodology:

" To create a controlled burn injury, we used the method described by Faunce et al. [14]. The mice were anesthetized with isoflurane, 55.5 mg/kg of ketamine Intra peritoneal (lP), and 2.6 mg/kg of xylazine IP (Webster Veterinary, Sterling, MA). While anesthetized, each mouse's dorsum was shaved. Each mouse was then placed individually in a plastic template designed to expose 15% total body surface area (TBSA) of skin calculated as described 16. Mice were then exposed to 94 degrees Celsius (for the burn groups) or room temperature water baths (for the sham groups) for 8 seconds as previously described [14,15]. Following this method, exposure of the mice's dorsum to 94 degrees Celsius water induces a localized full-thickness skin burn [14]. After this procedure, each mouse received additional pain medication (0.13 mg/kg buprenorphine IP) and normal saline IP for resuscitative purposes according to the parkland formula (4 x body weight (kg) x TBSA(%)). The mice were assessed and monitored through the post-procedure recovery preserving normothermia using warm pads."

2. Line 94, The animal model has been well established following Dr. Plackett Studies since 2003, which initially studied immunity response after burn injury. Are there any rationales of choosing two strains of mice to conduct the validation in this study? Also, the model was based on female mouse. Would authors comment whether the gender difference affects the data interpretation in the burn animal model?

Author's response: Thank you for the observation. We noticed in the current literature that liver function and cytochrome activity could differ in each mouse strain (1). We wanted to correlate our transcriptomic findings with two of the most wildly used mouse strains (blabl/c and C57BL/6) to evaluate whether the hepatic response to burns is strain-specific. However, we are not aware of any publications that directly compare the response of C57BL/6 mice and BALB/c mice to skin burn injury in the liver that we could directly compare our results with.

In terms of sex differences, among human according to the American Burn Association (2), women have higher mortality rates after burn than men even thought in general their have lower risk of developing life-threatening complication after trauma, which makes them good candidates for studying liver response to injury as a potential explanation of those discrepancies. As sex differences were not the objective of this study we decided to focus on one sex, but we do recognize that as a limitation of our study. We added this issue to the limitations paragraph at the end of the discussion section.

"This study was performed using female mice, given that publications demonstrate higher mortality rates in women who sustain burn injuries than men; however, sex differences can impact the results in murine models of burn injuries [58-60]"

1. Rudeck J, Bert B, Marx-Stoelting P, Schönfelder G, Vogl S. Liver lobe and strain differences in the activity of murine cytochrome P450 enzymes. Toxicology. 2018 Jul 1;404:76-85.
2. American Burn Association. National burn repository 2019 update: Report of data from 2009–2018. American Burn Association: Chicago, IL, USA. 2019:60606.
3. Haider AH, Crompton JG, Oyetunji T, Stevens KA, Efron DT, Kieninger AN, et al. Females have fewer complications and lower mortality following trauma than similarly injured males: a risk adjusted analysis of adults in the National Trauma Data Bank. Surgery. 2009;146(2):308–15

3. Line 98, the condition of food intake with food component could be added since the study explores the metabolic changes in mice.

Author's response: We added this sentence to the methods section:

"Mice were maintained on a 12-hour light/dark cycle with lights on at 8:00 am. All animals were kept in cages with controlled temperature (23°C-24°C) and humidity (60% ± 10%). The mice were fed with certified standard chow and tap water ad libitum."

4. Line 103, please check the typo "so."

Author's response: It was corrected.

5. Line 109, 1ml of saline was applied IP on burned mice. Have authors considered the different amount of resuscitation fluid required in young and aged mice after receiving 15% tbas burn? Does the burn animal receive the sufficient saline for resuscitation?

Author's response: We appreciate the reviewer's observation. After burn injury in our murine model, we administer IP fluid resuscitation following the parkland formula (4 x body weight (kg) x TBSA(%)) as in other studies (1). We corrected the previous statement in the methods section.

1. Hernandez A, Patil NK, Bohannon JK. A Murine Model of Full-Thickness Scald Burn Injury with Subsequent Wound and Systemic Bacterial Infection. Methods Mol Biol. 2021; 2321: 111–120.

6. Line 112, please describe euthanasia method.

Author's response: We modified the following sentence to describe the euthanasia method.

"Lastly, the mice were humanly euthanized using CO_2 followed by cervical dislocation, 24 hours (for transcriptomics and metabolomics data) and at multiple points 6-, 9-,12-, and 24 hours in a time course experiment (for transcriptomics validation) after the burn injury procedure."

7. Line 119, please provide RNA extraction/quality method, and qPCR method.

Author's response: We added the following paragraph to the manuscript:

"**Gene expression analysis**

RNA was extracted from liver using a RNeasy mini kit (Qiagen, Hilden, Germany) as previously described [9-11], following the manufacturer's recommended protocol. cDNA was then synthesized using an iScript kit according to manufacturer protocol (Bio-Rad, Hercules, CA). Quantitative RT-PCR was was performed using Universal SYBR Green Supermix (BioRad, #1725124). Standard desalted primers were purchased from Sigma. Cyp2c29 Forward Primer: 5' GCTCTCCTACTCCTGCTGAAGT 3' Reverse Primer: 5' ATGTGGCTCCTGTCTTGCATGC 3' Cyp2c38 Forward Primer: 5' CCTTGTCCCTAACAACCTACCC 3' Reverse Primer: 5' GGAACTCCTTGCTGTCATGCAG 3' Cyp2c40 Forward Primer: 5' CAAGAGGAAGCACAGTGGCTCA 3' Reverse Primer: 5' GGAAAACAATGGAGCAGATGACAT 3' Cyp2c54 Forward Primer: 5' GGACATCTGCCAATCCTTCACC 3' Reverse Primer: 5' GGTCAACCAGAGCTTCCTTCAC 3' run on a QuantStudio 3 Real-Time PCR System (Applied Biosystems, Foster City, CA). Gene expression was quantified using the ΔΔCt algorithm, with Gapdh as the endogenous control (Cat No. 4352339E, Applied Biosystems, Foster City, CA)."

8. Line 142, qPCR validation were taken 160 mice total in the study. please justify the rationale of the animal amount based on the animal experiment 3Rs principle.

Author's response: We are sorry for this inconsistency. One hundred mice instead of 160 were used for validation purposes because, for Balb/c mice, only one timepoint was evaluated. Regarding 3Rs principles, we could not replace animals in this experimental setting with *in vitro* culture as we need the whole organism's response to skin injury and its effect on remote liver tissues. For reduction, using 5 animals per experimental group was a crucial minimum to achieve statistical significance for highly variable genes like Cyp (please look at the SEM in the graphs for young sham mice). As we did not see many changes in early time points (6h, 9h, and 12h) in the second strain of animals, we only examined 24h time points to reduce the number of animals. Regarding refinement, animals were anesthetized during the procedure, later received pain medication and fluid therapy, and recovered with warm pads.

9. Line 363, please clarify if "b57bl" is a typo.

Author's response: It was corrected.

Reviewer #2 (Remarks to the Author):

In this manuscript, Malachowska et al., analyzed transcriptomics and metabolomics data from the liver of young and aged mice suffering burn-induced trauma. The study highlights pathway interactions and master regulators that underlie the liver response to burn injury in young and aged mice. Also, computer-based in-silico analysis was conducted to predict therapeutic targets to prevent or reverse burn-induced liver damage.
Although the reviewer agrees with the authors that the work will add some significant knowledge to the understanding of the pathophysiology of burn-induced trauma from the perspective of the liver, the study is in some aspects somewhat preliminary. In particular, the conclusions for the therapeutic targets to prevent or reverse burn-induced liver damage is somewhat questionable. The following issues should be addressed prior publication.

Author's response: Yes, we agree with the reviewer that our results are more a preliminary screening that definitely needs further validation. The main objective of our manuscript was to use bioinformatic approach to derive a new strategy or concept for treating burn injury. We modified a sentences in discussion to address this issue:

“However, the recommendation of using any of the predicted medications in a clinical scenario needs to be supported by in-depth in vivo drug studies and clinical trials as our results have a preliminary character. More follow-up studies are needed to validate the concept and develop a new treatment for burn injury patients.

Major issue:
1. Young and old BALB/c and C57BL/6 female mice were used in the study. Why were only female mice were used? The authors should at least explain the reasons in the Method section.

Author's response: Thank you so much for this comment. However, we are not aware of any publications that directly compare the response of C57BL/6 mice and BALB/c mice to skin burn injury in the liver that we could directly compare our results with.

In terms of sex differences, among human according to the American Burn Association (2), women have higher mortality rates after burn than men even thought in general their have lower risk of developing life-threatening complication after trauma, which makes them good candidates for studying liver response to injury as a potential explanation of those discrepancies. As sex differences were not the objective of this study we decided to focus on one sex, but we do recognize that as a limitation of our study. We added this issue to the limitations paragraph at the end of the discussion section.

“This study was performed using female mice, given that publications demonstrate higher mortality rates in women who sustain burn injuries than men; however, sex differences can impact the results in murine models of burn injuries [58-60]”

1. Rudeck J, Bert B, Marx-Stoelting P, Schönfelder G, Vogl S. Liver lobe and strain differences in the activity of murine cytochrome P450 enzymes. Toxicology. 2018 Jul 1;404:76-85.
2. American Burn Association. National burn repository 2019 update: Report of data from 2009–2018. American Burn Association: Chicago, IL, USA. 2019:60606.

3. Haider AH, Crompton JG, Oyetunji T, Stevens KA, Efron DT, Kieninger AN, et al. Females have fewer complications and lower mortality following trauma than similarly injured males: a risk adjusted analysis of adults in the National Trauma Data Bank. Surgery. 2009;146(2):308–15

2. The immune responses of BALB/c mice and C57BL/6 mice are very different. Why were two mice strains used and were these equally distributed between the groups?

Can the authors provide some relevant evidence that these two mice respond differently or similarly to burn-induced injury at the transcriptomic and metabolomic level in liver? Were there any strain differences noted in the analyses performed?

Author's response: BALB/c and C57BL/6 mice have different immune systems. According to Trunova et al. 2011 "*In BALB/c mice, volume density of T zone in the spleen and production of IL-2, IL-3, IL-4, IL-10, and TNF-α were much higher than in C57Bl/6 mice. However, IL-12 production in BALB/c mice was lower than in C57Bl/6 mice. C57Bl/6 mice were characterized by the higher cytostatic activity of splenic NK cells."* As the immune system is responsible for transmitting the information from the injury site to the liver to orchestrate a coordinated inflammatory response, it is safe to assume that those differences can contribute to a different response to burn injury in those two mice strains. To examine if the observed in our transcriptomic data difference are also present in another strain, we performed validation measurements with both C57Bl/6 and BALB/c mice. However, the qPCR analysis (Fig. S1) showed the similar trend between BALB/c and C57BL/6 mice. All omics experiments were performed in one strain for coherence. We added the following sentence in the methods section:

"All omics experiments were performed on BALB/c mice, and only for qPCR validation, we used both strains."

3. During trauma, large quantities of immune cells accumulate in the liver. Some of the upregulated genes found in this study are also marker genes of myeloid cells (such as S100a9, S100a8). The changes observed by the authors might not be restricted to responses in hepatocytes and might be due to the massive recruitment of immune cells, particularly myeloid cells. Was histology performed to evaluate for leukocyte infiltration differences?

Author's response: Thank you for the comment. Hepatic infiltration of neutrophils is an acute response to recent or ongoing liver injury, hepatic stress, or systemic inflammatory signals. While inflammatory cells increase significantly within the hepatic parenchyma, hepatocytes remain the prevalent cell type constituting 80% of the entire cell population (1,2,3). Thus whole liver transcriptomics is still relevant to obtain general hepatocyte gene expression data. However, we acknowledge that Immune cells in the liver can influence the liver transcriptome by producing cytokines and other signaling molecules that affect gene expression in hepatocytes and other liver cells. Inflammatory cytokines produced by immune cells can induce the expression of genes involved in inflammation and immune response in hepatocytes. Although whole liver transcriptomics can help study hepatocytes, it is essential to carefully interpret the data in the liver microenvironment context and consider other approaches, such as isolating hepatocytes, to obtain a more specific view of hepatocyte-specific gene expression patterns.

In the study by Li et al., liver samples one day after burn injury were compared. No significant increase of neutrophils infiltration was observed as well as no increase in MPO activity which a marker of neutrophils (4).

We added this observation to the limitations paragraph at the end of the discussion section.

“Since this study used the whole liver for transcriptomics and metabolomics analysis, we recognize that other parenchymal and immune cells in the liver can greatly impact the liver transcriptome/metabolome, which should be considered when analyzing this data. However, other studies showed that one day after skin burn injury, there is no significant increase of neutrophils infiltration MPO activity in liver samples [57].”

1. Gao B, Jeong WI, Tian Z. Liver: an organ with predominant innate immunity. Hepatology. 2008 Feb;47(2):729-36.
2. Yang W, Tao Y, Wu Y, Zhao X, Ye W, Zhao D, Fu L, Tian C, Yang J, He F, Tang L. Neutrophils promote the development of reparative macrophages mediated by ROS to orchestrate liver repair. Nature communications. 2019 Mar 6;10(1):1076.
3. Morgan KT, Jayyosi Z, Hower MA, Pino MV, Connolly TM, Kotlenga K, Lin J, Wang M, Schmidts HL, Bonnefoi MS, Elston TC. The hepatic transcriptome as a window on whole-body physiology and pathophysiology. Toxicologic pathology. 2005 Jan;33(1):136-45.
4. Li X, Akhtar S, Kovacs EJ, Gamelli RL, Choudhry MA. Inflammatory response in multiple organs in a mouse model of acute alcohol intoxication and burn injury. J Burn Care Res. 2011 Jul-Aug; 32(4): 489–497.

4. Description of liver sample collection require more information. Many details are missing, such as, the method of perfusion; red blood cells removal process before RNA extraction. 5. How was liver sampling standardized?

Author's response: Thank you for the observation. We added more detail to the methods section addressing the reviewer's concerns.

“Following euthanasia, the livers of all mice were infused with ice-chilled saline via the portal vein. For consistency, the right hepatic lobe in each animal was snap frozen in liquid nitrogen and then stored at −80°C for further analysis. Livers were maintained in tubes on dry ice when pieces were removed for homogenization, and homogenized tissue was kept in tubes on ice prior to analysis.”

6. Supplemental figures were not uploaded and accessible.

Author's response: We are very sorry for that. We double check this time, and supplementary figure was included in the merged .pdf file so we hope it will be accessible to the reviewer. All supplementary tables were provided as ‘Data Set’ file.

7. The authors carry out a limited attempt at validation by running qPCR for Cyp2c. To markedly enhance the value of their screening study, they should determine if cytochrome p450 function is impaired in older mice after burn injury. This would validate the value of the screening approach.

Author's response: As a response to reviewer comment we contact an expert in cytochrome measurement – Jed Lampe to help us address this issue. We agree with the reviewer's suggestion that it would be helpful to determine whether the function of the cytochrome P450 cyp2c enzymes, whose expression was demonstrated to be downregulated in Figure S1, is decreased. Unfortunately, there is very close functional homology between all of the mouse cyp2c P450's, and no cyp isoform-specific substrates have been identified for the individual enzymes. This makes it impossible to measure the enzymatic activity of the specific enzymes in question. Even a "general" cyp2c substrate probe would unlikely generate interpretable data due to the varying expression levels of the various cyp2c enzyme isoforms. However, in response to the reviewer's comment, we would like to note that a very good correlation between cyp mRNA level and enzyme expression and activity exists for the mouse cyp enzymes (1), which may alleviate some concerns regarding enzyme activity and mRNA levels.

1. Hart SN, Cui Y, Klaassen CD, Zhong XB. Three patterns of cytochrome P450 gene expression during liver maturation in mice. Drug Metab Dispos. 2009 Jan;37(1):116-21. doi: 10.1124/dmd.108.023812. Epub 2008 Oct 9. PMID: 18845660; PMCID: PMC2683655.

8. Methods of the 'in silico' analysis and other analysis is needed.

Author's response: Following paragraph was added to the manuscript:

"**In silico analysis**
To perform a functional analysis, construct networks, and predict upstream regulators, we used QIAGEN's Ingenuity® Pathway Analysis (IPA®, QIAGEN Redwood City, www.qiagen.com/ingenuity). A network of direct and indirect connections of differentially expressed genes and metabolites overlapping for young and aged mice after burn injury was used to feed the IPA algorithm. Nodes without any connections were excluded from the analysis. Prediction of upstream regulators of metabolic and transcriptomic changes after the burn injury was performed only using experimentally proven data for hepatocytes and/or liver from Qiagen knowledge base.
In silico drug, repurposing prediction was made with the Connectivity map platform (https://clue.io/) 27. The platform correlates the observed gene set dysregulation with known effects of perturbagens on multiple cell lines. We narrowed our analysis to drugs tested on HepG2 cell lines as the closest to our experimental setting. For our analysis threshold for tau score >95 was considered to select drugs with similarity signature to our query, and <-95 for selecting drugs with the opposing signature. Tau score is a standardized measure ranging from -100 to 100, corresponding to the fraction of reference gene sets with more significant similarity to the drug than the current query. "

Minor issue:
1. In Figure 1B and figure legends, the number of mice in each group is not noted or described.

Author's response: The numbers were added to the figure captions accordingly.

2. Insertion of references is not standardized.

Author's response: Thank you for the comment and we have made the correction.

REVIEWERS' COMMENTS:

Reviewer #1 (Remarks to the Author):

Reviewer appreciates authors' replies and has no more questions.

Reviewer #2 (Remarks to the Author):

The authors were able to address my concerns to my satisfaction.

REVIEWERS' COMMENTS:

Reviewer #1 (Remarks to the Author):

Reviewer appreciates authors' replies and has no more questions.

Reviewer #2 (Remarks to the Author):

The authors were able to address my concerns to my satisfaction.

Authors' response: We kindly thank both reviewers for their time and all suggestions that help us improve our manuscript.
